# A 21-year dataset (2000–2020) of gap-free global daily surface soil moisture at 1-km grid resolution

**DOI:** 10.1038/s41597-023-01991-w

**Published:** 2023-03-15

**Authors:** Chaolei Zheng, Li Jia, Tianjie Zhao

**Affiliations:** State Key Laboratory of Remote Sensing Science, Aerospace Information Research Institute, Chinese Academy of Sciences, Beijing, 100101 China

**Keywords:** Hydrology, Hydrology

## Abstract

Global soil moisture estimates from current satellite missions are suffering from inherent discontinuous observations and coarse spatial resolution, which limit applications especially at the fine spatial scale. This study developed a dataset of global gap-free surface soil moisture (SSM) at daily 1-km resolution from 2000 to 2020. This is achieved based on the European Space Agency - Climate Change Initiative (ESA-CCI) SSM combined product at 0.25° resolution. Firstly, an operational gap-filling method was developed to fill the missing data in the ESA-CCI SSM product using SSM of the ERA5 reanalysis dataset. Random Forest algorithm was then adopted to disaggregate the coarse-resolution SSM to 1-km, with the help of International Soil Moisture Network *in-situ* observations and other optical remote sensing datasets. The generated 1-km SSM product had good accuracy, with a high correlation coefficent (0.89) and a low unbiased Root Mean Square Error (0.045 m^3^/m^3^) by cross-validation. To the best of our knowledge, this is currently the only long-term global gap-free 1-km soil moisture dataset by far.

## Background & Summary

Soil moisture (SM) is a key state variable in the climate system and hydrological cycle, and it controls the exchange of water, energy, and carbon fluxes between the land surface and atmosphere^1–6^. SM datasets are essential for a wide range of applications in hydrology, meteorology, climatology, and water resource management^7–13^. SM presents high spatial and temporal variability due to the complex interactions among various correlated variables such as soil texture and structure, topographic features, land cover patterns, vegetation properties, and meteorological forcing^14–17^. These factors generally are difficult to isolate, and their coupled impacts on SM variability vary significantly over time and space domain^13,18,19^.

Different ground observation techniques have been developed to measure SM, e.g., the gravimetric methods, time/frequency domain reflectometry, neutron probes, electrical resistivity measurements, heat pulse sensors, fiber optic sensors^2,8,11,18^. Based on these point-scale ground observations, global SM networks, such as the International Soil Moisture Network (ISMN), have been established, and significant progress has been made in characterizing the spatial and temporal variation of SM to improve our understanding of the earth system^20^. However, *in-situ* measurements are limited in terms of spatial representativeness, and extrapolation of such point-scale measurements to large spatial scale is usually complex and time-consuming, especially on land surface with high spatial heterogeneity^15,21–24^. Spatial and temporal quantification of SM distributions at regional and global scales based on these ground-observed datasets remains challenging^25^.

Satellite remote sensing technology can obtain surface SM (SSM) from regional to global scale, and the observations from active and passive microwave sensors are considered to be one of the best tools for SSM retrieval^16,26,27^. Various satellites and algorithms have been developed with the ability to map SSM from satellite-based microwave sensors^28^, such as the Advanced Microwave Scanning Radiometer–EOS (AMSR-E) and its successor AMSR2, Fengyun-3 MicroWave Radiation Imager (MWRI), the advanced scatterometer (ASCAT), Soil Moisture and Ocean Salinity (SMOS), Soil Moisture Active Passive (SMAP), and European Space Agency Sentinel-1 satellite^29–33^, and global soil moisture products have been produced accordingly^34–39^. Although significant progress has been made to merge various satellites data to improve the remote sensing SSM coverage, there are still many gaps in the daily SSM dataset due to the limited satellite orbit/swath and retrieving capability. For example, it is found that the widely used multi-sensor fusion SSM product from European Space Agency - Climate Change Initiative (ESA-CCI) product has a very low spatial coverage (roughly 20%) in the central and western Tibetan Plateau^40^.

Another key limitation is that most of these global SSM products are at relative coarse spatial resolution, e.g., tens of kilometers, which limits the applications in regional hydrological and agricultural studies. Several downscaling approaches have been proposed to improve the spatial resolution of SSM product by considering the impacts of different environmental variables^25^. The idea behind these downscaling methods is to establish either a statistical function or a physically based model between coarse-resolution SSM and fine-resolution auxiliary variables^41–47^. However, several limitations were found for these downscaling methods, including the linear or nonlinear assumption to define the impact of spatial heterogeneity^42–44^, the error of input fine-resolution data, the uncertainties associated with the model parameter estimates^45–47^, and these may introduce large uncertainty. These downscaling methods generally use complex and computationally intensive disaggregation algorithms that are generally unsuitable for a global implementation due to complex and varying nonlinear relationships between soil moisture and the determinant variables used for downscaling^48^. Consequently, the high-resolution SSM dataset with global coverage is still lacking. Machine learning algorithms are increasingly used to extract patterns and insights of different geospatial variables from the ever-increasing stream of Earth system science data^49^, and have been proved to be a feasible method to disaggregate SSM (capture the complex nonlinear relationships) at coarse resolution hence to generate high resolution SSM at global scale^50–55^.

Considering the importance of high-resolution gap-free SSM data, this study aims at generating a high-resolution SSM dataset at global scale with continuity at both space and time scales by developing a SSM downscaling algorithm based on machine learning method. A global gap-free SSM dataset at daily scale and 1-km spatial resolution from 2000 to 2020 is finally generated.

## Methods

### Experimental design

**Fig. 1:**
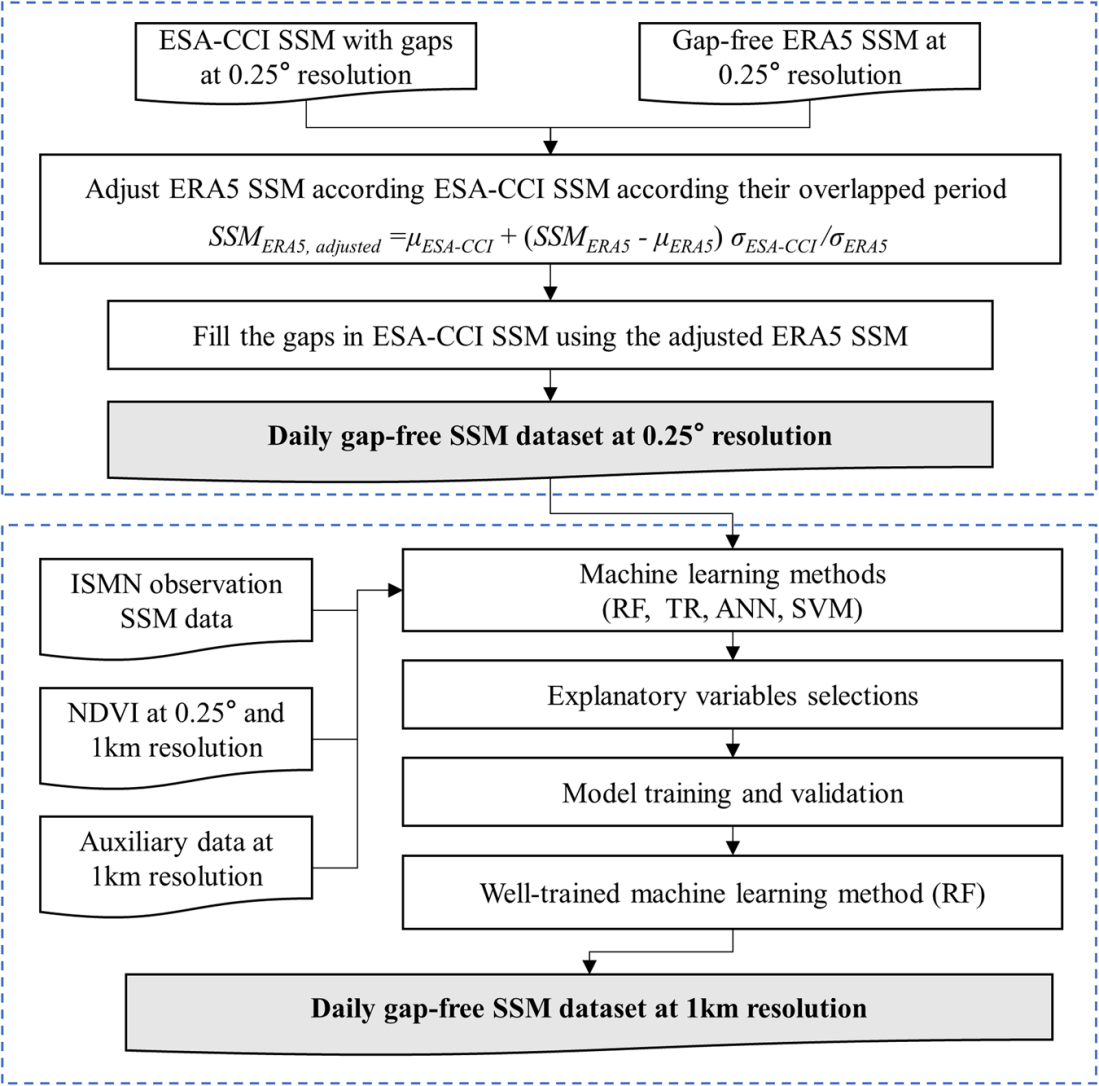
Schematic overview of the methodology and data products for generating the daily gap-free SSM dataset at 1-km resolution.

Previous studies on the evaluation of different soil moisture products^56–58^ conclude that ESA-CCI SSM has high accuracy and shows the best consistence with the ground observations. The top layer SM data from the European Centre for Medium-Range Weather Forecasts reanalysis v5 (ERA5) product shows good temporal correlation with ground observation, but with systematically large bias^57^. Hence, it’s reasonable to merge these two datasets by utilizing the high accuracy of ESA-CCI SM and good temporal variation and global gap-free coverage of ERA5 SM to generate a gap-free SSM dataset. The global daily gap-free SSM dataset at 1-km resolution was achieved by the following two steps in this study (Fig. 1). First, the ESA-CCI SSM product was gap-filled using ERA5 reanalysis product, and we achieved a daily gap-free SSM data at 0.25° resolution. Then, machine learning models were trained to downscale the daily gap-free SSM data at 0.25° resolution to 1-km resolution with the help of fine-resolution auxiliary data. We will introduce the data and algorithm used in this study in the following sections.

### Satellite and auxiliary data

**Fig. 2:**
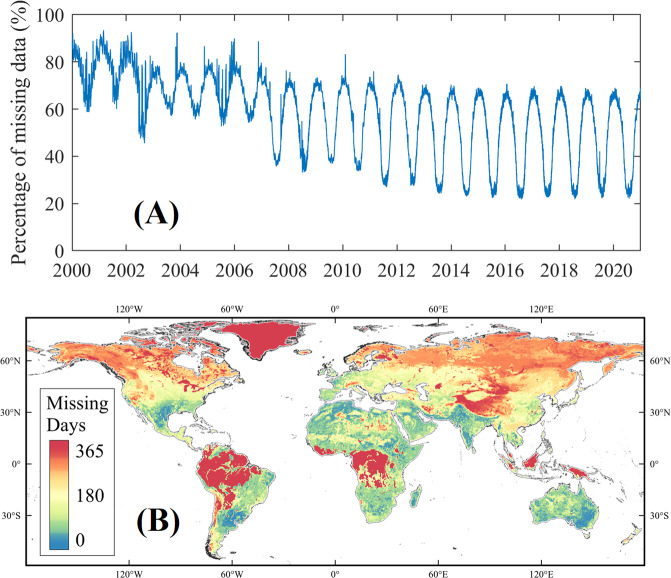
Statistics of missing data in the original ESA-CCI SSM products. (**A**) Percentage of missing data globally in ESA-CCI SSM dataset from 2000 to 2020. (**B**) Missing days per year in the original ESA-CCI SSM dataset averaged over multi-years (from 2000 to 2020).

The ESA-CCI SSM product is currently the available dataset of satellite-based soil moisture with the longest data record to date. The ESA-CCI SSM v06.1 product at coarse resolution was adopted in current study for further gap-filling and downscaling. The ESA-CCI SSM products was obtained using various satellite-based observations from microwave sensors since 1978, with 0.25° grid resolution at daily interval. It provides three SSM datasets: the merged dataset from observations by active microwave sensors (“Active Product”), the merged dataset from observations by passive microwave sensors (“Passive Product”), and the combined dataset. The “Active Product” and the “Passive Product” were created by fusing soil moisture products from scatterometer and radiometer observations, respectively. The combined SM product was obtained by merging all active and passive SSM observations directly through temporal resampling, spatial resampling, Cumulative Distribution Function (CDF) -based rescaling, and triple collocation analysis-based merging algorithm. We selected the combined dataset in this study since it was supposed to inherit the advantages of both active and passive microwave observations, and it generally outperformed the products using single-sensor observation as input^59^. Although enormous efforts have been conducted to obtain SSM at daily scale with global coverage, the daily ESA-CCI SSM products still could not fully cover the global land surface. The missing data percentage range from 21.8% to 94.41% at daily step from 2000 to 2020 according to our statistics, with an averaged value of 58.17% (Fig. 2). Even the global missing data percentage decreased dramatically with the increase of available satellite data after 2007, the minimum missing values of daily ESA-CCI SSM could still count for 21.8% of global land surface (Antarctica excluded). (Fig. 2). Large gaps are especially in winter time for the northern hemisphere due to frozen water content in soil, which is difficult to be detected by microwave bands^60,61^. Studies intended to exclude the densely vegetated regions, since the retrieval errors are relatively large in these regions because the sensitivity of the radiometer to SSM is reduced due to the strong attenuation of the ground emission signal by vegetation^62^. Missing data days per year were also high in high elevations regions, e.g., the missing observation days was found larger than 200 days per year in the Tibetan Plateau.

The top layer SM data from the ERA5 product was downloaded from the Copernicus Climate Data Store (https://cds.climate.copernicus.eu/) and used to fill the missing values in ESA-CCI SSM dataset. The ERA5 was built upon its predecessor (ERA-Interim), and it combined more historical observations and run on finer resolutions^63^. The ERA5 SSM has a globally spatial-temporal continuous coverage, with a spatial resolution of 0.25° and temporal resolution of 1 hr. In this study, daily ERA5 SSM was calculated by averaging the hourly ERA5 SSM. The ERA5 SSM has better performance in terms of correlation with ground observations than some other soil moisture reanalysis products^56^, and it could reasonably regenerate the monthly dynamics and annual cycles, especially the timings of the strong dry-wet transition^57^.

The predictors used in the machine learning algorithm for downscaling SSM includes Normalized Difference Vegetation Index (NDVI), surface albedo, digital elevation model (DEM), and saturated soil moisture. To obtain the 1-km resolution SSM data, different optical remote sensing datasets at high resolution were collected and processed to obtain daily values of these predictor variables at 1-km resolution. The monthly NDVI data with 1-km resolution were from MOD13A2 product^64^. The monthly 0.05° resolution NDVI from MOD13C1^65^ were used and aggregated to 0.25° resolution to match the spatial resolution of ESA-CCI SSM data in the downscaling model. They were further interpolated linearly to daily temporal resolution to match the temporal resolution of SSM. The albedo data was from the Global LAnd Surface Satellite (GLASS) product^66,67^, and it was reconstructed to daily step by linear interpolation for further application. The topographic information was retrieved from the SRTM30 DEM^68^, and the DEM at 1-km resolution was retrieved from its native 30 Arc Seconds resolution using bilinear interpolation method. The global 1-km saturated soil moisture was obtained from previous study that produce a high-resolution global map of soil hydraulic properties by a hierarchical parametrization of a physically based water retention model^69^, using the surface soil of SoilGrids dataset as input^70^.

### Ground observation data

The *in-situ* soil moisture observations datasets from ISMN were collected to train the machine learning method for SSM downscaling and validate the results. The ISMN is a soil moisture dataset network established and maintained through international cooperation. The SM observations have been collected around the world by different research teams and harmonized to make the data available for public research, through the coordination by the Global Energy and Water Exchanges Project^20^. To date, the ISMN data consists of measurements from 2,879 sites in 68 networks (last access on February 10, 2022). These *in-situ* SM observations play an increasingly substantial role in evaluating satellite and model products^2,20,71–74^. The ISMN data adopted in current study is listed in Supplementary Table S1. Further details about the instruments and the data quality control of the observations can be found in the network reports and references therein (available from https://ismn.geo.tuwien.ac.at).

### ESA-CCI SSM gap-filling at 0.25° resolution

The applicability of SSM data is often hindered by spatiotemporal gaps. Global reanalysis data are featured by high spatial coverage and high temporal resolution, which could be used to fill the gaps in remote sensing SSM dataset. However, in many regions, global reanalysis data lack of accuracy and are biased from ground true or satellite retrievals^62,64,74^. One solution is to make use of the consistency of temporal variation between the remote sensing SSM time series and the SSM of reanalysis data, while re-scale (or adjust) the magnitude of the reanalysis SSM according to the remote sensing SSM. In current study, the missing values in ESA-CCI SSM was gap-filled by using the ERA5 SSM to obtain daily gap-free ESA-CCI SSM at 0.25° resolution. To avoid the inconsistency between the ESA-CCI SSM and the ERA5 SSM, the daily ERA5 SSM was re-scaled (adjusted) according to the ESA-CCI SSM before it was used for gap-filling. The re-scaling of ERA5 SSM was done by establishing a linear relationship for each 0.25° grid between these two SSM time series using daily data on the overlapped days. A simple linear relationship between the ERA5 SSM and ESA-CCI SSM on overlapped days for each 0.25° grid could be built as below

1 $$SS{M}_{ESA-CCI}=a\,SS{M}_{ERA5}+b$$

where *a* and *b* were fitting parameters. Once the coefficients *a* and *b* are defined in Eq. (1), the re-scaled (or adjusted) ERA5 SSM, *SSM*_*ERA5, adjusted*_, could be obtained as

2 $$SS{M}_{ERA5,adjusted}=a\,SS{M}_{ERA5}+b$$

Assuming the adjusted daily ERA5 SSM and the original daily ERA5 SSM depart the same way from their mean values of time series (*μ*_*ESA-CCI*_ and *μ*_*ERA5*_) with the same standard deviations (*σ*_*ESA-CCI*_ and *σ*_*ERA5*_), the following equations could be obtained,

3a $$SS{M}_{ERA5,adjusted}-{\mu }_{ESA-CCI}=SS{M}_{ERA5}-{\mu }_{ERA5}$$

3b $$\left(SS{M}_{ERA5,adjusted}-{\mu }_{ESA-CCI}\right)/{\sigma }_{ESA-CCI}=\left(SS{M}_{ERA5}-{\mu }_{ERA5}\right)/{\sigma }_{ERA5}$$

Equation (3b) could be rearranged as

4 $$SS{M}_{ERA5,adjusted}={\sigma }_{ESA-CCI}/{\sigma }_{ERA5}\cdot SS{M}_{ERA5}+{\mu }_{ESA-CCI}-{\mu }_{ERA5}\cdot {\sigma }_{ESA-CCI}/{\sigma }_{ERA5}$$

Hence, the pixel-wise *a* and *b* in Eq. (2) were obtained, and *SSM*_*ERA5, adjusted*_ was estimated using the averaged values and standard deviation values of ERA5 SSM and ESA-CCI SSM over the overlapped days. *SSM*_*ERA5, adjusted*_ estimated by Eq. (4) was then adopted to fill the missing values in the ESA-CCI SSM for each 0.25° grid. For the 0.25° grids where no overlapped data were found (roughly 10% of global land surface), mainly in the tropical rainforest regions, the ERA5 SSM was directly adopted to fill the missing values in the ESA-CCI SSM dataset. Even it may be controversy, ERA5 also gives the SSM value for the water and snow/ice covered pixels, and it was directly used to fill the missing values of water and snow/ice covered pixels during the SSM gap-filling phase in this study. Finally, the global daily gap-free SSM at 0.25° resolution was achieved.

### Spatial downscaling

The daily gap-free ESA-CCI SSM at 0.25° resolution was disaggregated to 1-km using machine learning method. The disaggregation strategy first learns the nonlinear relationships between the *in-situ* observations of SSM and the ESA-CCI soil moisture at 0.25° resolution and NDVI at both 0.25° and 1-km resolutions, to predict the fine resolution (1-km) SSM. The ISMN observation data were adopted to train the machine learning model for SSM downscaling. We also tested the performance of different machine learning methods and different combinations of explanatory variables, which allowed us to select the most suitable model and explanatory variables for SSM downscaling.

We first explored the performances of using different explanatory variables, several tests were conducted with different variables included in the machine learning models (Table 1). The *SSM*_*25km*_, *NDVI*_*25km*_ and *NDVI*_*1km*_ was employed in Test1. The surface albedo (*α*_1km_), digital elevation model (*DEM*_*1km*_), and surface saturated soil moisture (*θ*_*S,1km*_), were added successively from Test2 to Test8. These variables were selected because of their physical relationships with the spatial variation of SSM. For instance, high SSM values generally associated with good vegetation conditions (high NDVI and low albedo). The NDVI both at coarse and fine resolutions were used in previous studies^51^. The albedo showed exponential relationship with SSM and systematic decrease in albedo in response to rainfall were observed widely^75,76^. *DEM*_*1km*_ and *θ*_*S,1km*_ somehow related to the soil water holding capacity^40^. Land surface temperature (LST) was highly related to SSM, but it was not selected for SSM downscaling since the gap-free LST at moderate (e.g., 1-km) resolution were not available due to the impact of cloudiness. It’s also noted that precipitation was not selected as an explanatory variable considering that the global moderate resolution precipitation dataset was not available. We will consider to update the auxiliary datasets in the future when they are available.

**Table 1:** Experiment design to explore the performances by using different explanatory variables for SSM downscaling.

Test	Explanatory variables
Test1	SSM_25km_, NDVI_25km_, NDVI_1km_
Test2	SSM_25km_, NDVI_25km_, NDVI_1km_, *α*_1km_
Test3	SSM_25km_, NDVI_25km_, NDVI_1km_, DEM_1km_
Test4	SSM_25km_, NDVI_25km_, NDVI_1km_, *θ*_*S, 1km*_
Test5	SSM_25km_, NDVI_25km_, NDVI_1km_, *α*_1km_, DEM_1km_
Test6	SSM_25km_, NDVI_25km_, NDVI_1km_, *α*_1km_, *θ*_*S, 1km*_
Test7	SSM_25km_, NDVI_25km_, NDVI_1km_, DEM_1km_, *θ*_*S, 1km*_
Test8	SSM_25km_, NDVI_25km_, NDVI_1km_, DEM_1km_, *α*_1km_, *θ*_*S, 1km*_

We tested and compared four machine learning algorithms to select the most accurate algorithm for SSM downscaling, including the Random Forest (RF), the Support Vector Machine (SVM), the Tree-based Regression (TR), and the Artificial Neural Networks (ANN). We used *k*-fold (*k* = 10 in current study) cross validation to validate and compare the downscaled SSM by different models and tests. The SSM observation data from ISMN was divided into k-fold (or groups) randomly, with one of the folds (10% of the observation data) was left as ‘unmeasured’ and the remaining *k*-1 folds (90% of the observation data) were used for training the models. The trained models were validated based on the ‘unmeasured’ data. The training and validation procedures were repeated 10 times, using a different fold as the holdout set for each time; hence all data was selected for validation. This validation could explore the transferability of the downscaling model from known *in-situ* SSM observation sites to any other sites for global applications. The correlation coefficient (*R*) and unbiased root-mean-square error (*ubRMSE*) were used to evaluate the performances of the four different machining learning methods.

**Fig. 3:**
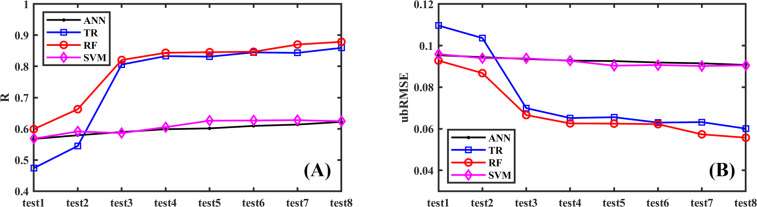
Comparison of the performance of SSM_1km_ predictions based on the four machine learning models using different explanatory variables.

Figure 3 illustrated the performance of different machine learning models using different combinations of explanatory variables by cross validation. Generally, with the inclusion of more explanatory variables in all the four models, *R* was increasing, and *ubRMSE* was decreasing. Best performance (with high *R* and low *ubRMSE*) was found in Test8, when all six explanatory variables were selected. This indicated that the SSM can be predicted accurately when all the selected explanatory variables were included in the downscaling model. Hence, all six explanatory variables (*SSM*_*25km*_, *NDVI*_*25km*_, *NDVI*_*1km*_, *DEM*_*1km*_, α_1km_, *θ*_*S, 1km*_) were adopted in the SSM downscaling model. All these four machine learning algorithms showed good performance in predicting SSM (R > 0.6 for all). The Tree and RF methods showed much better performance than ANN and SVM, the best results were given by the RF method with the highest *R* (0.89) and lowest *ubRMSE* (0.05 m^3^/m^3^) than the other three algorithms (Fig. 3). Based on the performance analyzed above, the RF model was applied to downscale SSM product and to generate global daily/1-km resolution SSM product using the explanatory variables in Test8, i.e., SSM and NDVI at 25-km resolution, NDVI, surface albedo, DEM and surface saturated soil moisture at 1-km resolution.

## Data Records

The final daily/1-km SSM product accounts for more than 1 TB of data capacity. Due to the storage limitation of the online repositories, we provide the monthly averaged 1-km SSM data for download from the data portal of National Tibetan Plateau/Third Pole Environment Data Center^77^ (10.11888/RemoteSen.tpdc.272760) after user registration. Data are freely available in this data portal. For easy read and manipulation, the monthly 1-km SSM data are stored in geotiff format with one file for each month with global coverage. Users can use most Geographic Information Systems (GIS) and remote sensing software packages to read and manipulate the data. The file names follow the structure of “SM.1km.Month.YYYYMM.Global.v001.tif”, where “SM.Month.1km.” represents the 1-km monthly averaged SSM product, “YYYY” is the year, “MM” represents the month, “Global” represents the global coverage, and “v001” indicates the product version.

Daily SSM in customized regions are available on request to the corresponding author (zhengcl@aircas.ac.cn) with details of the intended and desired spatial and temporal resolution, domain, and period of interest in user’s request. The detailed information of the daily SSM product is shown in Table 2. The daily 1-km SSM data are stored in hdf5 format (https://www.hdfgroup.org/), and global data was divided into tiles with sinusoidal grid projection following the data structure of MODIS products. Each file covers roughly 10° × 10° area, and more details on the tiles and projection could be seen from https://modis-land.gsfc.nasa.gov/MODLAND_grid.html. The file names of daily 1-km SSM data follow the structure of “SM.1km.Daily.YYYYDOY.tiles.v001.h5”, where“SM.Daily.1km” represents the daily 1-km SSM product,“YYYY” is the year,“DOY” represents the day of the year (from 001 to 365 or 366),“tiles” represents the tile number (e.g., h24v05) according to the MODIS sinusoidal grid, and “v001” indicates the product version. A quality flag was provided in the daily SSM dataset, ranging from 0 to 8 (0: original ESACCI SSM was used; 1: original ESA-CCI SSM was not available and the gap-filled SSM was used; 2 ~ 3: RF algorithm was failed, and value was from simple linear or nearest interpolation; 4 ~ 7: input data was missing, e.g. albedo, NDVI; 8: non-soil pixels). It should be noticed that SSM retrieval/downscaling is not available for water and snow/ice surfaces, and those pixels with NDVI below zero were set as non-soil pixels and marked in quality flag for the 1-km SSM. Users can use Python, IDL, MATLAB, etc., to read and manipulate the data.

**Table 2:** Detailed information about the Scientific DataSet in the daily SSM product.

Data	Long name	Number type	Unit	Fill value	Scale factor	Added offset	Valid range
SM	Daily soil moisture	Int16	m^3^/m^3^	−1	0.001	0	0 ~ 1000
flag	Flag of daily soil moisture	Int8	None	−1	none	none	0 ~ 8

## Technical Validation

### Validation of the gap-filled SSM at resolution of 0.25°

**Fig. 4:**
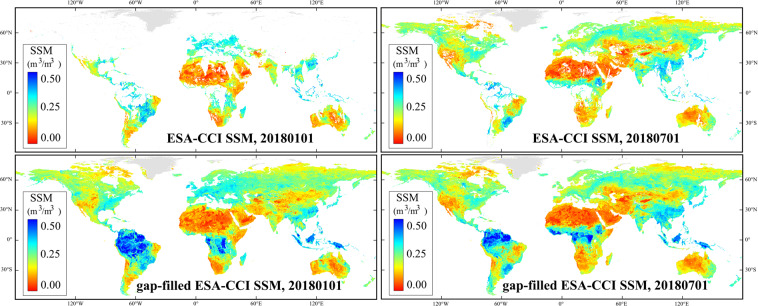
Global map examples of the original ESA-CCI SSM product and gap-filled ESA-CCI SSM on January 1^st^ and July 1^st^, 2018 (m^3^/m^3^).

Theoretically, the percentage of SSM data gaps can be reduced to zero after the gap-filling procedure in this study. Figure 4 shows the examples of the gap-filled SSM on typical winter and summer days. Given that SSM does not apply to non-soil pixels such as water and snow/ice cover surfaces, these pixels are masked in Fig. 4. Generally, the gap-filled SSM data could capture the global SSM spatial variation while retaining the original information of ESA-CCI SSM.

**Fig. 5:**
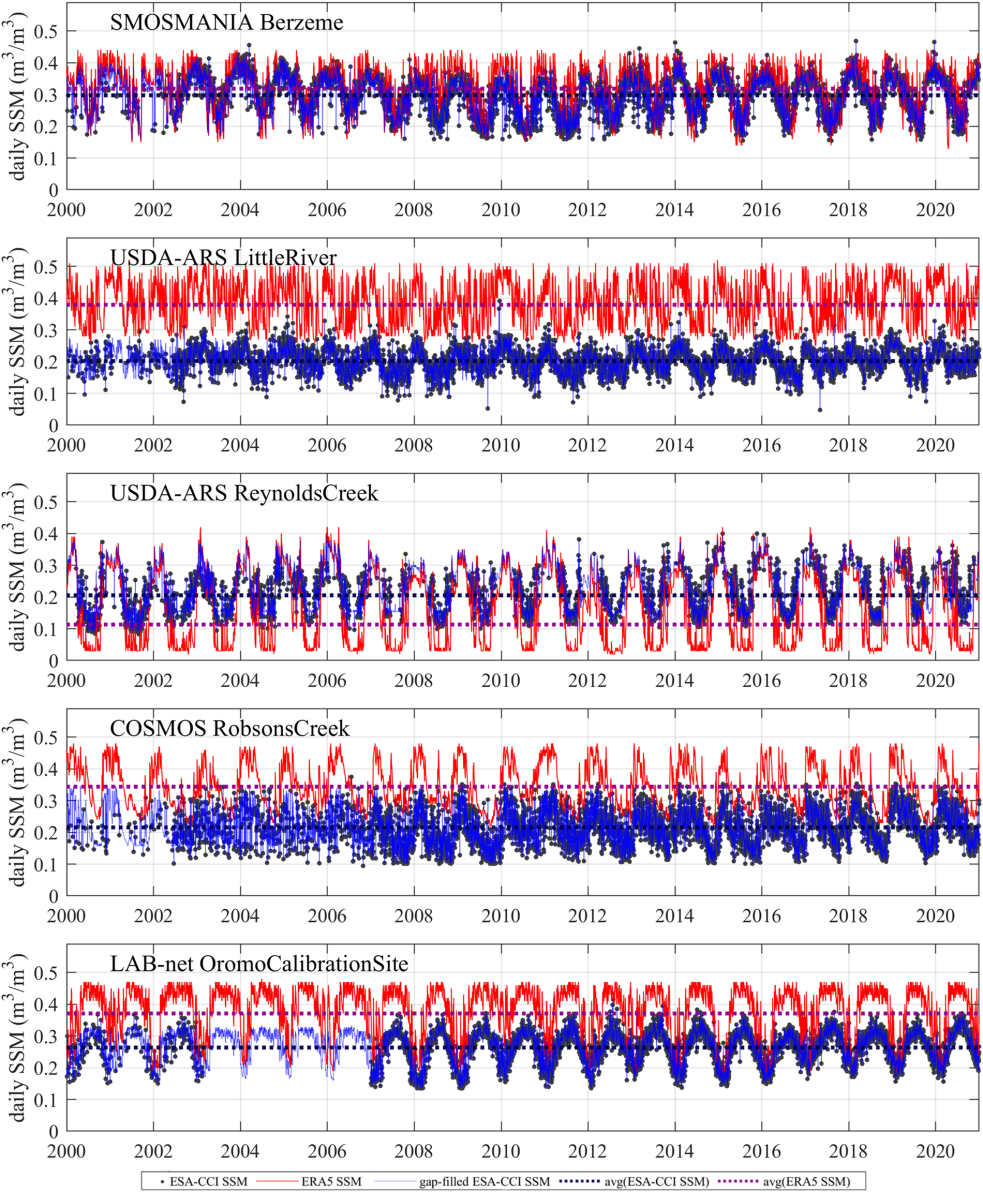
Examples of the time series of the gap-filled ESA-CCI SSM and the original ESA-CCI SSM product, ERA5 SSM products in 0.25° grids at the selected ISMN sites in 2000–2020.

Figure 5 represents the temporal behaviours of the original ESA-CCI SSM and the gap-filled ESA-CCI SSM in 0.25° grids at selected ISMN sites. Large gaps could be found in the early years of the time series of the original ESA-CCI SSM in some selected grids. The average and standard deviation values of ESA-CCI SSM and ERA5 SSM during their overlapped days are also shown in Fig. 5. Although relatively large difference could be found between ESA-CCI SSM and ERA5 SSM in some sites grids, their temporal variations are generally consistent with each other. The gap-filled ESA-CCI SSM followed the temporal variation of ERA5 SSM and original ESA-CCI SSM, and showed consistent magnitude with the original ESA-CCI SSM. The averaged values of gap-filled ESA-CCI SSM are also stable, and systematic error introduced by the gap-filling method is negligible.

**Fig. 6:**
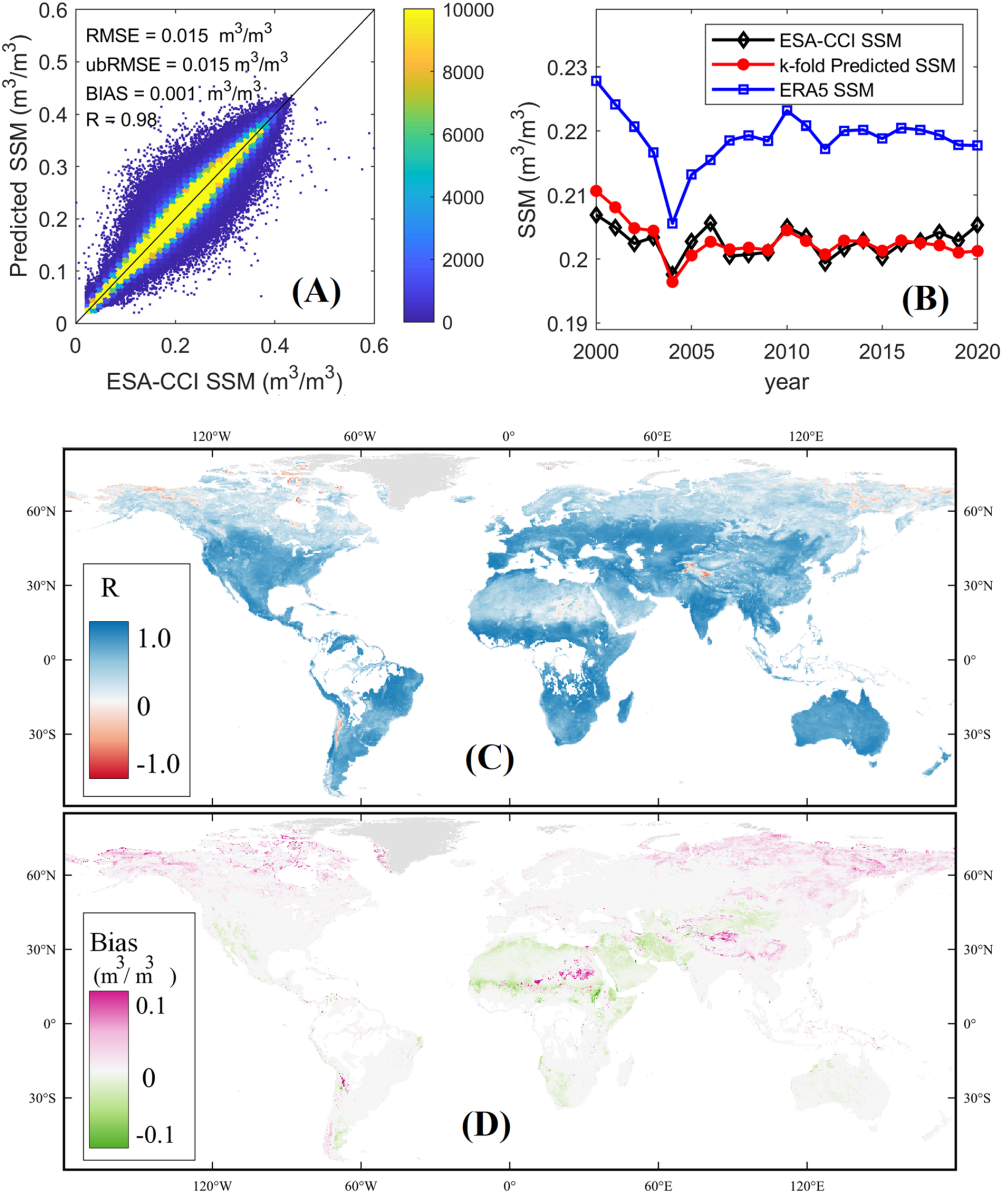
Results of *k*-fold cross-validation of the gap-filled ESA-CCI SSM: (**A**) Scatterplot of the predicted ESA-CCI SSM against the original ESA-CCI SSM; (**B**) Temporal variation of annual mean values of the predicted ESA-CCI SSM, original ESA-CCI SSM, and ERA5 SSM; (**C**) *R* of predicted ESA-CCI SSM, (**D**) Bias of predicted ESA-CCI SSM.

To validate the reasonability of the gap-filling method, a *k*-fold (*k* = 10 in current study) validation was conducted. The daily ESA-CCI SSM values in each 0.25° grid in 2000–2020 were randomly divided into 10 folds, and each fold of the 10-folds was taken out and predicted by the remaining 9 folds. This procedure was repeated 10 times until all the 10 folds of ESA-CCI SSM data were traversed (predicted). All the predicted SSM values were gathered to compare with the original ESA-CCI SSM series, the results were shown in Fig. 6. It generally shows the reliability of the gap-filled ESA-CCI SSM with overall high *R* values (0.98) and low bias (0.001 m^3^/m^3^) globally between the gap-filled ESA-CCI SSM and the original ESA-CCI SSM (Fig. 6A). The temporal variation of annual global mean values of the predicted SSM showed very close pattern to the original ESA-CCI SSM (Fig. 6B). Lower *R* was found in high-latitude cold regions in the northern hemisphere and the extreme arid region (e.g. the Sahel desert and the western Tibetan Plateau) (Fig. 6C). There were more missing data in the ESA-CCI SSM dataset in the high-latitude cold regions in the northern hemisphere, which partly explained the lower *R* and larger bias (Fig. 6C and Fig. 6D). In the desert regions, SSM was very low, and low *R* was anticipated due to larger uncertainty in the retrieved SSM under very dry condition.

### Validation of the downscaled SSM at 1-km resolution based on ISMN observation

**Fig. 7:**
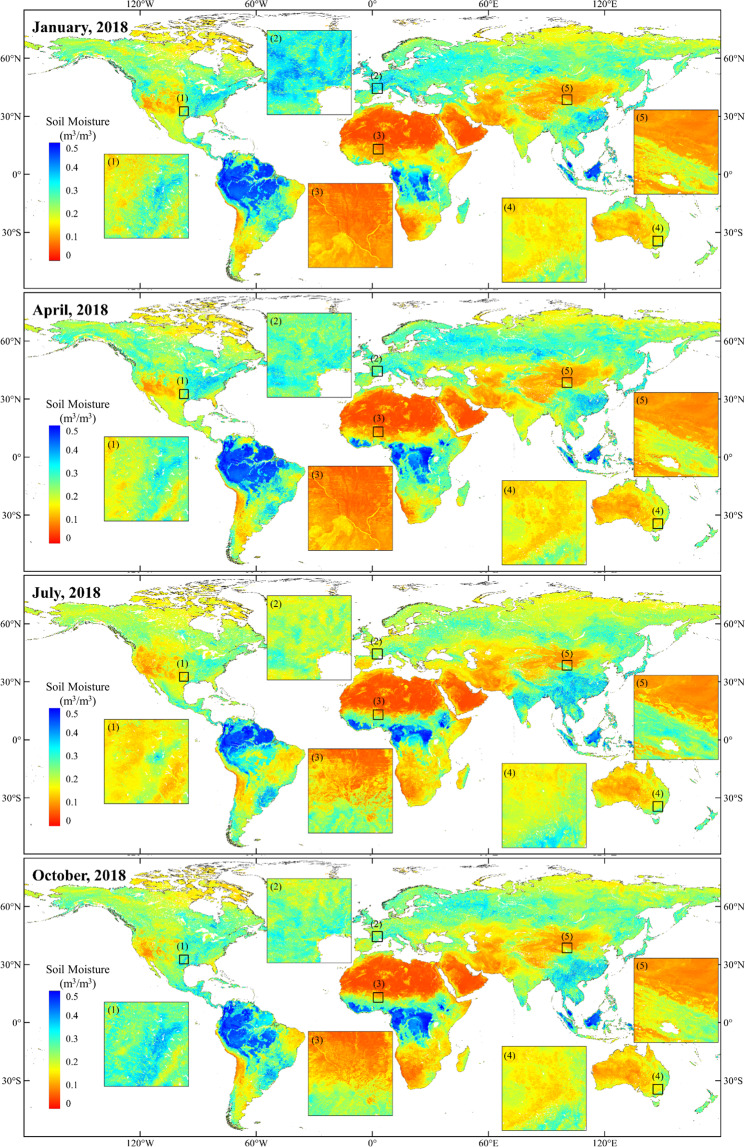
Global distribution of monthly averaged SSM at 1-km resolution in January, April, July and October of 2018. The zoom-in views of the selected sub-regions cover different ISMN networks: (1) USDA-ARS network of America, (2) SMOSMANIA network in Europe, (3) AMMA-CATCH network in Africa, (4) OZNET network in Australia, and (5) HiWATER-EHWSN in China.

As demonstrated in the Method section, the RF model outperformed other methods, it was therefore used to produce global daily/1-km SSM from 2000 to 2020. Figure 7 presents some examples of monthly averaged downscaled SSM at 1-km resolution in January, April, July, and October of 2018. For better illustration of the regional SSM distribution, zoom-in views of different selected sub-regions are also shown in Fig. 7. The selected sub-regions in Fig. 7 cover roughly 5° × 5° with ISMN network included, which are 1) USDA-ARS network of America, 2) SMOSMANIA network in Europe, 3) AMMA-CATCH network in Africa, 4) OZNET network in Australia, and 5) HiWATER-EHWSN in China. The global 1-km SSM can capture well the overall spatial variations of global SSM, and the spatial features of SSM are well illustrated by the high-resolution SSM as shown in the sub-region maps.

Supplementary Table S2 lists the accuracy metrics of the downscaled 1-km SSM in each ISMN network compared with the ISMN observations. The overall bias of the downscaled 1-km SSM is 0 m^3^/m^3^ with the range from −0.065 to 0.015 m^3^/m^3^, *R* is 0.89 with the range from 0.325 to 0.962, *ubRMSE* is 0.045 m^3^/m^3^ with the range from 0.015 m^3^/m^3^ to 0.069 m^3^/m^3^.

**Fig. 8:**
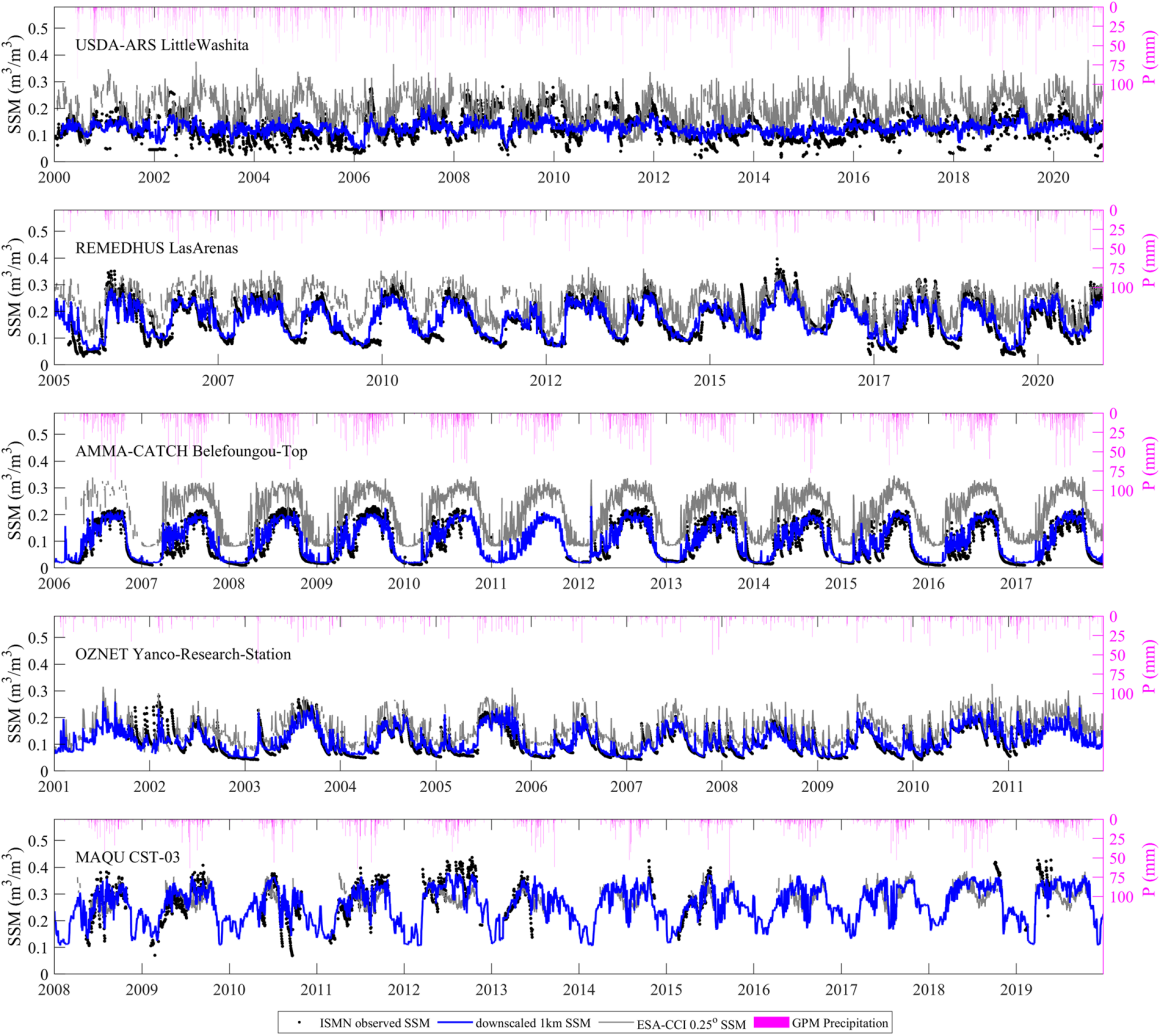
Time series of the downscaled 1-km SSM from the grids at the selected ISMN sites. Precipitation (P) from GPM product was also shown.

Figure 8 shows the temporal variation of downscaled 1-km SSM at selected ISMN sites. The 1-km SSM are very close to the ground observations, and it can trace the seasonal variation of SSM very well. Although the ground measured data used in Fig. 8 were included in training the models (hence not independent), it is acceptable to take them as reference for evaluation of the performance of the downscaled SSM. The results demonstrated the prediction ability of the RF model used for downscaling of SSM. Meanwhile, to illustrate the ability of the 1-km soil moisture data to detect rainfall events, precipitation information was also shown in Fig. 8. Clearly, the soil moisture fluctuates with precipitation, especially in the arid land, e.g., the OZNET Yanco-Research station, where the dry-down process (soil moisture depletes following the precipitation event) could be well captured by the 1-km soil moisture data.

## Usage Notes

In this study, we provided a dataset of global spatiotemporally continuous daily surface soil moisture at 1-km resolution from 2000 to 2020 for various applications and studies. The 1-km SSM dataset generated in this study has several potential applications. For example, it was successfully applied to ETMonitor model for global high-resolution evapotranspiration estimation^78^, in which soil moisture was used to parametrize soil surface resistance to soil evaporation and canopy resistance to plant transpiration, so as to better consider the influence of soil moisture on land surface evapotranspiration. Compared with those evapotranspiration dataset that did not use soil moisture information, e.g., the MOD16 evapotranspiration product, the error of estimated evapotranspiration by ETMonitor decreased significantly after using SM information at 1-km resolution, e.g., RMSE of global 8-days evapotranspiration decreased from 1.34 mm/d by MOD16 to 0.83 mm/d by ETMonitor^78^.

The high-resolution SSM dataset also has the potential in distinguishing irrigated fields, inferring irrigation water use, improving wildfire danger prediction, etc. However, the size of farmland is generally small, and higher spatial resolution (e.g., 30-m) may be more appropriate for distinguishing irrigation and non-irrigation fields. Furthermore, it is not practical to assess the performance of identifying irrigation fields using the 1-km SM data due to lack of *in-situ* irrigation information. Further evaluation will be needed to assess the capability of the downscaled 1-km SSM for distinguishing between irrigated and non-irrigated fields. An alternative way is that the irrigation information could be achieved by comparing satellite derived and modelled SM (the latter does not include irrigation information)^79^, or by inverting soil water balance equation to derive the total in-flow water in the soil^80^. However, we should notice these retrieve algorithms need to be calibrated carefully to achieve good accuracy^81^.

Notably, the 1-km resolution SSM in this study is obtained by downscaling the low-resolution SSM data, which is essentially to spatially redistributed microwave-based soil moisture in the coarse grid (0.25°) to enclosed pixels (grids) at high resolution (1-km in this study), hence the high-resolution SSM inherits the uncertainty of the low-resolution SSM product. Although comparison with the *in-situ* observations from the ISMN at global scale shows satisfactory accuracy, considering that the *in-situ* observation sites used for validation are relatively sparse and the distribution of ISMN is extremely uneven, it is impossible to guarantee the same quality in different regions of the world. Moreover, one should be aware of the limitation of machine-learning-based model, which cannot always correctly capture the variations in SM. Previous study has reported that machine-learning-based model failed to track the ‘tipping points’ (where a slowly changing soil moisture triggered a sudden shift to a new soil moisture) when applied to SSM prediction^82^. In additions to the inherent capability of machine-learning-based models, the choice of explanatory variables has a significant impact on the results, and the uncertainty in the employed input datasets will certainly be propagated into the downscaled 1-km SSM. The temporal resolution of the dataset is achieved by our method using ERA5 dataset for day-by-day filling. However, it is difficult to analyse the actual physical spatial resolution, which can be very complex and related to all microwave and optical datasets used in the study. It can be inferred that the actual physical resolution would vary from location to location. Therefore, cautions should be paid when applying the downscaled SSM dataset for further analysis, e.g., for detection of convective rainfall events, for prediction of flood and landslide risk at high resolution.

All the monthly 1-km SSM data are stored in geotiff in the data portal of National Tibetan Plateau/Third Pole Environment Data Center^77^ (10.11888/RemoteSen.tpdc.272760). The daily 1-km SSM data, stored in hdf5 format, are available on request to the corresponding author (zhengcl@aircas.ac.cn). Users can freely choose the spatial and temporal coverage of SSM dataset according to their specific research objectives. Users can use Python, IDL, MATLAB, and popular Geographic Information Systems (GIS) or remote sensing software packages to read and manipulate the data. It should be noted that the data must be multiplied by their corresponding scale factors (in Table 2). Instructions for data post-processing (converting to geographic coordinates, etc.) is provided with the data upon request. The dataset will be updated in the future when new or better input data become available.

## Supplementary information

Supplementary Materials

## Data Availability

The codes used in this study will be available at https://github.com/zhengchaolei/GlobalSSMGapfillDownscaling.git after this work is accepted.
