# Supplementary material for: A 21-year dataset (2000–2020) of gap-free global daily surface soil moisture at 1-km grid resolution
Source: Sci Data. 2023 Mar 15;10:139. doi: 10.1038/s41597-023-01991-w (PMC10017679; doi:10.1038/s41597-023-01991-w)
Supplement: Supplementary file 1 — Supplementary Materials [file 41597_2023_1991_MOESM1_ESM.docx]

**Supplementary Materials for**

A 21-year dataset (2000-2020) of gap-free global daily surface soil moisture at 1-km grid resolution

### Authors

Chaolei Zheng ^1^*, Li Jia ^1^, Tianjie Zhao ^1^

**Affiliations**

1. State Key Laboratory of Remote Sensing Science, Aerospace Information Research Institute, Chinese Academy of Sciences, Beijing 100101, China

corresponding author(s): Chaolei Zheng (zhengcl@aircas.ac.cn)

Supplementary Table S1. Information of the adopted ISMN data

| **Network** | **Number of Sites** | **Start Year** | **End Year** | **Obs. Depths**  **(cm)** | **Sensors** |
| --- | --- | --- | --- | --- | --- |
| AACES | 49 | 2005 | 2010 | 5~6 | ThetaProbe-ML2X |
| AMMA-CATCH | 7 | 2006 | 2017 | 5 | CS616-1& CS616-2 |
| ARM | 35 | 2000 | 2020 | 5 | Hydraprobe-II |
| AWDN | 50 | 2000 | 2020 | 10 | ThetaProbe-ML2X |
| BIEBRZA_S-1 | 30 | 2015 | 2018 | 5 | GS-3 |
| BNZ-LTER | 12 | 2000 | 2020 | 5 | CS615 |
| COSMOS | 108 | 2010 | 2020 | 4~7 | Cosmic-ray-Probe |
| CTP_SMTMN | 57 | 2000 | 2020 | 5 | EC-TM |
| DAHRA | 1 | 2004 | 2016 | 5 | ThetaProbe-ML2X |
| AMERIFLUX | 8 | 2000 | 2020 | 2~5 | ThetaProbe-ML2X |
| FMI | 27 | 2000 | 2020 | 5 | ThetaProbe-ML2X |
| FR_Aqui | 5 | 2012 | 2020 | 3~5 | ThetaProbe-ML2X |
| GROW | 150 | 2017 | 2019 | 10 | Flower-Power |
| GTK | 7 | 2001 | 2012 | 10 | CS616 |
| HOAL | 33 | 2000 | 2020 | 5 | SPADE-TDT -A |
| HOBE | 32 | 2000 | 2020 | 5 | Decagon-5TE-A&B |
| HYDROL-NET_PERUGIA | 2 | 2010 | 2016 | 5 | TDR |
| HiWATER-EHWSN | 174 | 2012 | 2012 | 4 | Hydraprobe-II |
| ICN | 18 | 2000 | 2010 | 10 | Troxler-Neutron-Surface-Probe |
| IIT_KANPUR | 1 | 2011 | 2012 | 10 | WaterScout-SM100 |
| IPE | 2 | 2012 | 2020 | 6~10 | CS650 |
| KHOREZM | 7 | 2010 | 2011 | 5 | ThetaProbe-ML2X |
| KIHS_CMC | 18 | 2018 | 2019 | 10 | Buriable-Waveguide |
| KIHS_SMC | 19 | 2018 | 2019 | 10 | Buriable-Waveguide |
| LAB-net | 4 | 2014 | 2020 | 7 | CS616 |
| MAQU | 27 | 2008 | 2019 | 5 | ECH20-EC-TM |
| MOL-RAO | 2 | 2003 | 2020 | 8 | TRIME-EZ |
| MONGOLIA | 19 | 2000 | 2002 | 10 | Coring-device-auger |
| NAQU | 11 | 2010 | 2019 | 5 | 5TM |
| NGARI | 23 | 2010 | 2019 | 5 | 5TM |
| NVE | 3 | 2012 | 2019 | 10 | Delta-T-PR2-daily-averages |
| ORACLE | 6 | 2000 | 2020 | 5~6 | TRASE-16 |
| OZNET | 38 | 2001 | 2018 | 5~8 | CS615 |
| PBO_H2O | 158 | 2006 | 2017 | 4~5 | GPS |
| REMEDHUS | 24 | 2005 | 2020 | 5 | Stevens-Hydra-Probe |
| RISMA | 23 | 2000 | 2020 | 5 | Hydraprobe-II |
| RSMN | 20 | 2014 | 2020 | 5 | 5TM |
| Ru_CFR | 2 | 2000 | 2020 | 5 | Hydraprobe-II |
| SASMAS | 14 | 2005 | 2007 | 5 | CS616 |
| SCAN | 238 | 2000 | 2020 | 2.5~5 | Hydraprobe-Analog- |
| SD_DEM | 1 | 2005 | 2020 | 5 | CS616 |
| SMN-SDR | 34 | 2018 | 2019 | 5 | 5TM |
| SMOSMANIA | 22 | 2000 | 2020 | 5 | ThetaProbe-ML2X |
| SNOTEL | 438 | 2000 | 2020 | 5~7.6 | Hydraprobe-Analog |
| SOILSCAPE | 169 | 2011 | 2017 | 4~5 | EC5 |
| SW-WHU | 7 | 2014 | 2015 | 10 | LVDSC12 |
| SWEX_POLAND | 6 | 2000 | 2020 | 5 | D-LOG-mpts |
| TAHMO | 70 | 2019 | 2020 | 5 | TEROS12 |
| TERENO | 5 | 2000 | 2020 | 5 | Hydraprobe-II |
| UDC_SMOS | 11 | 2000 | 2020 | 5 | EC-ET& EC5 |
| UMSUOL | 1 | 2009 | 2017 | 10 | TDR-100 |
| USCRN | 115 | 2000 | 2020 | 5 | Stevens-Hydraprobe-II |
| USDA-ARS | 4 | 2000 | 2020 | 5 | Hydraprobe-Analog |
| VAS | 3 | 2010 | 2012 | 5 | ThetaProbe-ML2X |
| VDS | 4 | 2000 | 2020 | 1~10 | GS1-Port |
| iRON | 10 | 2000 | 2020 | 5 | EC5-II& EC5-I |

Supplementary Table S2. Statistical metrics of the performance of downscaled 1-km SSM compared with ISMN observations data.

| **Network** | **Number of Samples** | **Obs. SSMavg**  **(m^3^/m^3^)** | **Predict SSMavg**  **(m^3^/m^3^)** | **BIAS**  **(m^3^/m^3^)** | **ubRMSE**  **(m^3^/m^3^)** | **R** |
| --- | --- | --- | --- | --- | --- | --- |
| AACES | 163 | 0.151 | 0.130 | -0.020 | 0.050 | 0.890 |
| AMMA-CATCH | 12973 | 0.102 | 0.104 | 0.002 | 0.025 | 0.943 |
| ARM | 185320 | 0.174 | 0.175 | 0.000 | 0.052 | 0.820 |
| AWDN | 102872 | 0.179 | 0.178 | -0.001 | 0.043 | 0.902 |
| BIEBRZA_S-1 | 5958 | 0.333 | 0.328 | -0.005 | 0.025 | 0.898 |
| BNZ-LTER | 17018 | 0.202 | 0.201 | 0.000 | 0.046 | 0.865 |
| COSMOS | 9964 | 0.273 | 0.265 | -0.008 | 0.034 | 0.946 |
| CTP_SMTMN | 38120 | 0.174 | 0.175 | 0.001 | 0.053 | 0.876 |
| DAHRA | 1602 | 0.052 | 0.058 | 0.006 | 0.021 | 0.737 |
| AMERIFLUX | 16772 | 0.168 | 0.164 | -0.004 | 0.035 | 0.959 |
| FMI | 23131 | 0.187 | 0.188 | 0.001 | 0.055 | 0.834 |
| FR_Aqui | 5101 | 0.151 | 0.151 | 0.000 | 0.033 | 0.892 |
| GROW | 40297 | 0.248 | 0.231 | -0.017 | 0.064 | 0.711 |
| GTK | 4561 | 0.232 | 0.229 | -0.004 | 0.035 | 0.886 |
| HOAL | 116294 | 0.181 | 0.186 | 0.005 | 0.064 | 0.787 |
| HOBE | 125428 | 0.159 | 0.159 | 0.000 | 0.049 | 0.817 |
| HYDROL-NET_PERUGIA | 3482 | 0.236 | 0.230 | -0.006 | 0.030 | 0.880 |
| HiWATER_EHWSN | 15124 | 0.264 | 0.263 | -0.001 | 0.043 | 0.743 |
| ICN | 4332 | 0.302 | 0.291 | -0.011 | 0.049 | 0.831 |
| IIT_KANPUR | 462 | 0.185 | 0.178 | -0.007 | 0.035 | 0.949 |
| IPE | 3562 | 0.163 | 0.166 | 0.003 | 0.050 | 0.889 |
| KHOREZM | 87 | 0.218 | 0.182 | -0.036 | 0.069 | 0.407 |
| KIHS_CMC | 8875 | 0.213 | 0.213 | 0.000 | 0.035 | 0.720 |
| KIHS_SMC | 38 | 0.159 | 0.160 | 0.001 | 0.054 | 0.325 |
| LAB-net | 992 | 0.280 | 0.277 | -0.003 | 0.034 | 0.948 |
| MAQU | 27170 | 0.277 | 0.276 | 0.000 | 0.042 | 0.906 |
| MOL-RAO | 8509 | 0.112 | 0.114 | 0.002 | 0.028 | 0.916 |
| MONGOLIA | 657 | 0.272 | 0.207 | -0.065 | 0.052 | 0.651 |
| NAQU | 4947 | 0.127 | 0.128 | 0.001 | 0.029 | 0.901 |
| NGARI | 14919 | 0.084 | 0.085 | 0.001 | 0.019 | 0.905 |
| NVE | 1029 | 0.198 | 0.194 | -0.004 | 0.026 | 0.734 |
| ORACLE | 20085 | 0.191 | 0.192 | 0.000 | 0.057 | 0.778 |
| OZNET | 108800 | 0.137 | 0.137 | 0.000 | 0.033 | 0.915 |
| PBO_H2O | 171646 | 0.125 | 0.124 | -0.001 | 0.033 | 0.910 |
| REMEDHUS | 94280 | 0.126 | 0.127 | 0.001 | 0.029 | 0.948 |
| RISMA | 90418 | 0.181 | 0.181 | 0.000 | 0.050 | 0.796 |
| RSMN | 29716 | 0.144 | 0.150 | 0.007 | 0.027 | 0.892 |
| Ru_CFR | 8992 | 0.208 | 0.208 | 0.001 | 0.048 | 0.799 |
| SASMAS | 7581 | 0.154 | 0.160 | 0.006 | 0.044 | 0.924 |
| SCAN | 614087 | 0.186 | 0.186 | 0.000 | 0.044 | 0.921 |
| SD_DEM | 531 | 0.050 | 0.053 | 0.002 | 0.015 | 0.788 |
| SMN-SDR | 9562 | 0.166 | 0.165 | -0.001 | 0.032 | 0.914 |
| SMOSMANIA | 79961 | 0.197 | 0.197 | 0.000 | 0.040 | 0.892 |
| SNOTEL | 746316 | 0.169 | 0.169 | 0.000 | 0.049 | 0.889 |
| SOILSCAPE | 86591 | 0.154 | 0.154 | 0.001 | 0.049 | 0.864 |
| SW-WHU | 215 | 0.201 | 0.216 | 0.015 | 0.067 | 0.683 |
| SWEX_POLAND | 20637 | 0.136 | 0.138 | 0.002 | 0.047 | 0.793 |
| TAHMO | 6056 | 0.250 | 0.249 | -0.001 | 0.031 | 0.962 |
| TERENO | 30076 | 0.166 | 0.167 | 0.001 | 0.043 | 0.784 |
| UDC_SMOS | 68754 | 0.167 | 0.168 | 0.002 | 0.044 | 0.779 |
| UMSUOL | 2591 | 0.236 | 0.235 | -0.002 | 0.036 | 0.879 |
| USCRN | 428908 | 0.163 | 0.164 | 0.000 | 0.041 | 0.859 |
| USDA-ARS | 28107 | 0.121 | 0.123 | 0.002 | 0.034 | 0.749 |
| VAS | 1038 | 0.142 | 0.146 | 0.003 | 0.031 | 0.864 |
| VDS | 13589 | 0.117 | 0.119 | 0.002 | 0.041 | 0.727 |
| iRON | 17359 | 0.146 | 0.148 | 0.002 | 0.047 | 0.808 |
| **All data** | **3.49×10^6^** | **0.170** | **0.170** | **0.000** | **0.045** | **0.89** |
